# Authentic Phosphorylation of α‑Synuclein at Ser129 Reveals Functional Differences Not Captured by the S129D Phosphomimetic

**DOI:** 10.1021/acschembio.6c00118

**Published:** 2026-04-23

**Authors:** Scott G. Allen, Christopher Williams, Matthew P. Crump, Robert J. Williams, Jody M. Mason

**Affiliations:** † Department of Life Sciences, 1555 University of Bath, Bath BA2 7AY, United Kingdom; ‡ School of Chemistry, 1980 University of Bristol, Bristol BS8 1TS, United Kingdom

## Abstract

Phosphorylation at serine 129 (pS129) is a dominant post-translational modification of α-synuclein (αSyn) and a widely used pathological marker in Parkinson’s disease, yet its mechanistic consequences remain debated across physiological and pathological contexts. Most studies rely on phosphomimetic substitutions such as S129D, which approximate net charge but do not reproduce the steric, geometric, or hydrogen-bonding properties of authentic phosphorylation. Here, we establish a robust bacterial coexpression platform that generates homogeneous, site-specifically phosphorylated αSyn using its native kinase, Polo-like kinase 2. Using this system, we show that authentic pS129 differs fundamentally from S129D: it induces local, NMR-detectable perturbations within the C-terminal conformational ensemble, exhibits distinct and context-dependent aggregation behavior, and elicits neuronal responses and modest, reproducible toxicity not reproduced by phosphomimetics. These data resolve inconsistencies in the literature and highlight the importance of chemically authentic post-translational modification. More broadly, this platform provides a generalizable and scalable route to chemically faithful phosphorylated proteins, enabling more accurate interrogation of post-translationally regulated protein function.

## Graphical abstract

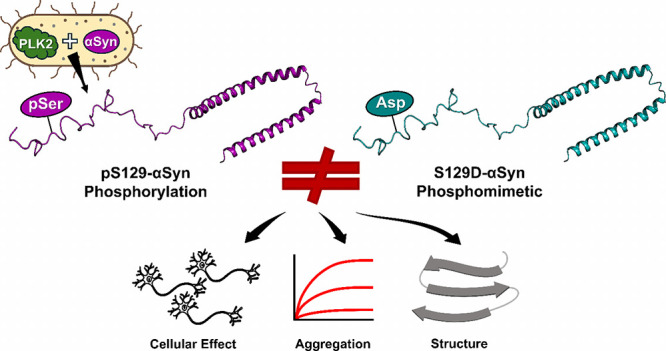

The pathological aggregation of α-synuclein (αSyn) into Lewy body inclusions is a defining hallmark of Parkinson’s disease (PD).^1^ Although the molecular steps leading to αSyn aggregation remain elusive, several intermediate species have been implicated in the process (Figure 1).^2^ Among these, phosphorylation at serine 129 (pS129) is particularly prominent: approximately 96% of αSyn in Lewy bodies is phosphorylated at this site, compared with only ∼4% in healthy neurons.^3^ Although it remains unclear whether phosphorylation precedes or follows aggregation, pS129 is widely used as a disease biomarker and is interpreted as a marker of neurodegeneration.^4–6^ Despite its prevalence and disease association, the precise structural and functional consequences of pS129 remain incompletely understood.^7–10^ Reports have variously proposed that pS129 can promote or inhibit aggregation, alter αSyn conformation, or influence toxicity and clearance.^5,11–13^ More recently, pS129 has also been implicated in the regulation of synaptic activity and trafficking.^13–15^ Notably, much of the mechanistic ambiguity surrounding pS129 arises from the widespread use of phosphomimetic substitutions, which reproduce charge but not the full chemical identity of phosphorylation. Importantly, phosphorylation at Ser129 has also been implicated in normal synaptic activity and membrane-associated αSyn function, complicating simple pathological interpretations.^16^

**Figure 1:**
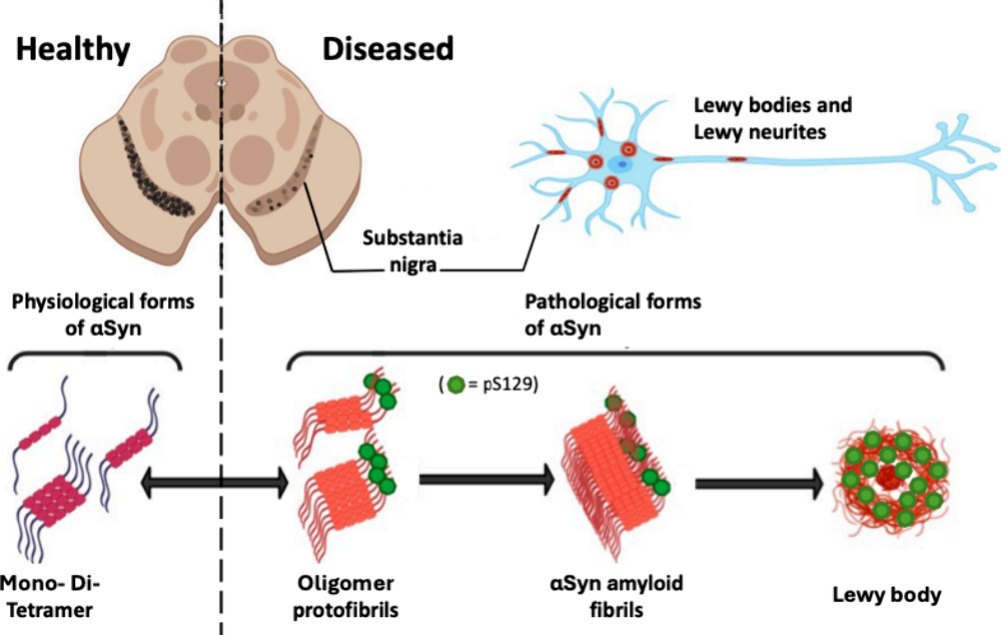
Pathological aggregation of αSyn in Parkinson’s disease (PD). In healthy neurons, αSyn exists primarily as a cytosolic monomer or tetramer with a disordered structure. In Parkinson’s disease, αSyn misfolds into β-sheet-rich structures, forming oligomers, protofibrils, and insoluble fibrillar aggregates. Phosphorylation at Ser129 (pS129), located at the C-terminus, is a major pathological hallmark, present in >96% of Lewy body inclusions. These pS129-positive aggregates accumulate in neuronal cell bodies (Lewy bodies) and neurites (Lewy neurites), particularly in the substantia nigra. Figure adapted from ref 21.

Despite the centrality of pS129 as a biomarker and potential contributor to pathology, most *in vitro* studies have relied on phosphomimetic substitutions, particularly S129D, as surrogates for authentic phosphorylation.^12,17^ These substitutions approximate the negative charge of a phosphate group but lack its steric and chemical complexity, raising fundamental concerns about the accuracy of mechanistic conclusions drawn from mimetic systems. This is reflected in inconsistent findings regarding the effects of S129D on aggregation kinetics and cellular toxicity, suggesting that such analogues may not faithfully recapitulate the functional outcomes of authentic phosphorylation.^17–20^ The absence of structurally faithful phospho-αSyn models represents a persistent limitation in studies of αSyn biology and disease, where structural fidelity to the native post-translationally modified protein is essential. Concerns regarding the validity of phosphomimetic substitutions at Ser129 have been raised for over a decade. Notably, Paleologou et al. demonstrated that authentic phosphorylation, but not S129D substitutions, suppresses αSyn fibrillation *in vitro*,^18^ highlighting fundamental chemical differences between phosphate and carboxylate modifications. More recent studies further emphasize that Ser129 phosphorylation is dynamically regulated, reversible, and closely linked to membrane-associated αSyn states, rather than serving as a simple pathological switch.^16^

To address these limitations, we developed a recombinant bacterial coexpression system in which αSyn is expressed alongside its native kinase, Polo-like kinase 2 (PLK2). This platform yields highly homogeneous, site-selectively phosphorylated pS129-αSyn suitable for direct comparison with both unmodified wild-type and phosphomimetic S129D variants. Using this system, we identify marked differences in NMR signatures, aggregation behavior, epitope recognition, and cellular responses. These data demonstrate that authentic phosphorylation introduces functional consequences that are not captured by phosphomimetics. Collectively, our findings offer new mechanistic insight into the role of pS129 in αSyn biology and establish a versatile tool for studying disease-relevant post-translational modifications. Unlike chemical or semisynthetic approaches, this method is economical, scalable, avoids hazardous solvents and expensive reagents, and yields fully recombinant, biologically relevant pS129-αSyn in a single step using its native kinase.

To enable site-specific phosphorylation of αSyn, we developed a PLK2 coexpression system that yields homogeneous Ser129-phosphorylated αSyn (pS129-αSyn). The enrichment of pS129-αSyn in PD highlights the need for reproducible, scalable access to this modified protein for mechanistic studies. αSyn is typically expressed in *E. coli* and purified using standard protocols, typically yielding highly pure protein.^22^ However, batch-to-batch variation and the absence of post-translational modifications limit the ability to interrogate phosphorylation-dependent effects with precision. To address this, we adapted a bacterial coexpression strategy in which αSyn is expressed together with its cognate kinase, PLK2,^11^ enabling efficient site-specific phosphorylation at Ser129 in a single recombinant step. PLK2 was selected because it is the dominant neuronal kinase responsible for S129 phosphorylation *in vivo*,^23^ yielding stoichiometric, site-specific modification without the mixed phospho-states or off-target modifications often observed in chemically or semisynthetically prepared material. The resulting pS129-αSyn is homogeneous, biologically relevant, and requires minimal downstream processing.

Mass spectrometry confirmed phosphorylation of αSyn by PLK2, with the phosphorylated variant displaying the expected +80 Da mass shift relative to wild-type protein (Figure 2a). Site-specificity was verified by NMR, where the ^1^H–^15^N heteronuclear single-quantum coherence (HSQC) spectrum of ^15^N-labeled pS129-αSyn revealed a characteristic downfield shift of the S129 resonance to ∼8.8 ppm (^1^H), accompanied by an upfield shift of the adjacent E130 residue, consistent with a localized, C-terminal structural reorganization centered on the phosphorylation site at S129 (Figure 2b). Immunoblotting using a pS129-specific antibody selectively detected the PLK2-phosphorylated protein, with no signal for wild-type or S129D variants (Figure 2c). Together, these data confirm that the coexpression platform yields homogeneous site-specifically phosphorylated pS129-αSyn suitable for direct comparison with unmodified and phosphomimetic forms. A faint secondary higher molecular weight band/species observed with pS129 may be caused by electrophoretic artifacts, or result from further, minor modifications. However, no secondary phosphorylation events were observed in the HSQC specifically on Ser or Thr residues (see also Supplementary Figure 1).

**Figure 2:**
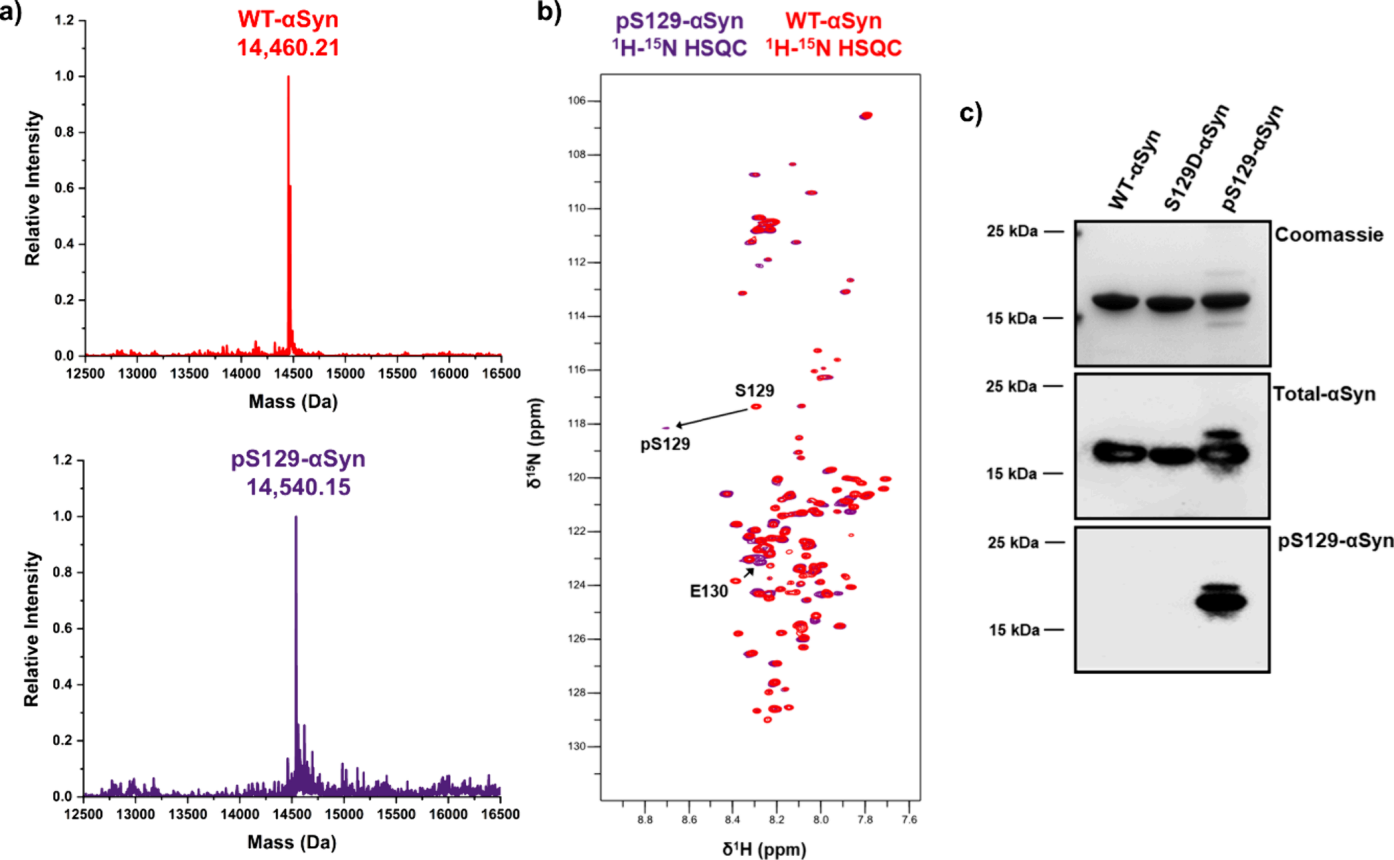
Characterization of site-specific phosphorylation of αSyn at Ser129 (pS129). (a) Deconvoluted ESI-MS spectra of purified wild-type αSyn (top, red) and αSyn coexpressed with PLK2 (bottom, purple). The phosphorylated variant shows a mass shift of +80 Da, consistent with single-site phosphorylation. (b) Overlaid ^1^H–^15^N HSQC spectra of ^15^N-labeled wild-type αSyn (red) and pS129-αSyn (purple) showing a downfield shift of the S129 amide resonance (∼8.8 ppm) and an upfield shift of E130, consistent with site-specific phosphorylation. Minor chemical shift perturbations localized around the S129 phosphorylation site are highlighted in Supplementary Figure 1. (c) Western blot analysis of wild-type, S129D, and pS129-αSyn probed with a pS129-specific antibody (Abcam EP1536Y) selectively recognizes the phosphorylated form. A pan-αSyn antibody (Cell Signaling Technology #2628) and Coomassie staining confirm total protein loading. Uncropped images are shown in Supplementary Figure 2.

We then examined the aggregation behavior of pS129-αSyn relative to wild-type and S129D variants. The impact of Ser129 phosphorylation on αSyn aggregation has remained unresolved, with reports describing both inhibitory^5^ and pro-aggregative^11^ effects depending on experimental conditions. These discrepancies may arise in part from the use of phosphomimetics, which differ chemically from authentic phosphorylation. To assess the influence of pS129 using fully defined protein preparations, we compared aggregation of wild-type αSyn, S129D-αSyn and authentic pS129-αSyn using both agitation-induced and lipid-induced conditions. Analytical SEC confirmed that αSyn variants were monomeric prior to initiation of aggregation assays (Supplementary Figure 4).

Under agitation, wild-type-αSyn exhibited the expected sigmoidal ThT fluorescence profile characteristic of fibril formation, whereas S129D-αSyn aggregated more rapidly but plateaued at a lower ThT intensity (Figure 3a). In contrast, pS129-αSyn showed minimal aggregation under these conditions (Figure 3a). CD spectroscopy confirmed a substantial β-sheet transition for both wild-type and S129D-αSyn, although the latter retained a residual negative band ∼190 nm, consistent with incomplete structural conversion (Figure 3b,c). In contrast, pS129-αSyn retained a disordered spectrum throughout the experiment (Figure 3d). These findings indicate that authentic phosphorylation biases the C-terminal conformational ensemble away from states that support long-range structural rearrangements associated with fibrillation, thereby limiting the transition to β-sheet-rich assemblies under these conditions.

**Figure 3:**
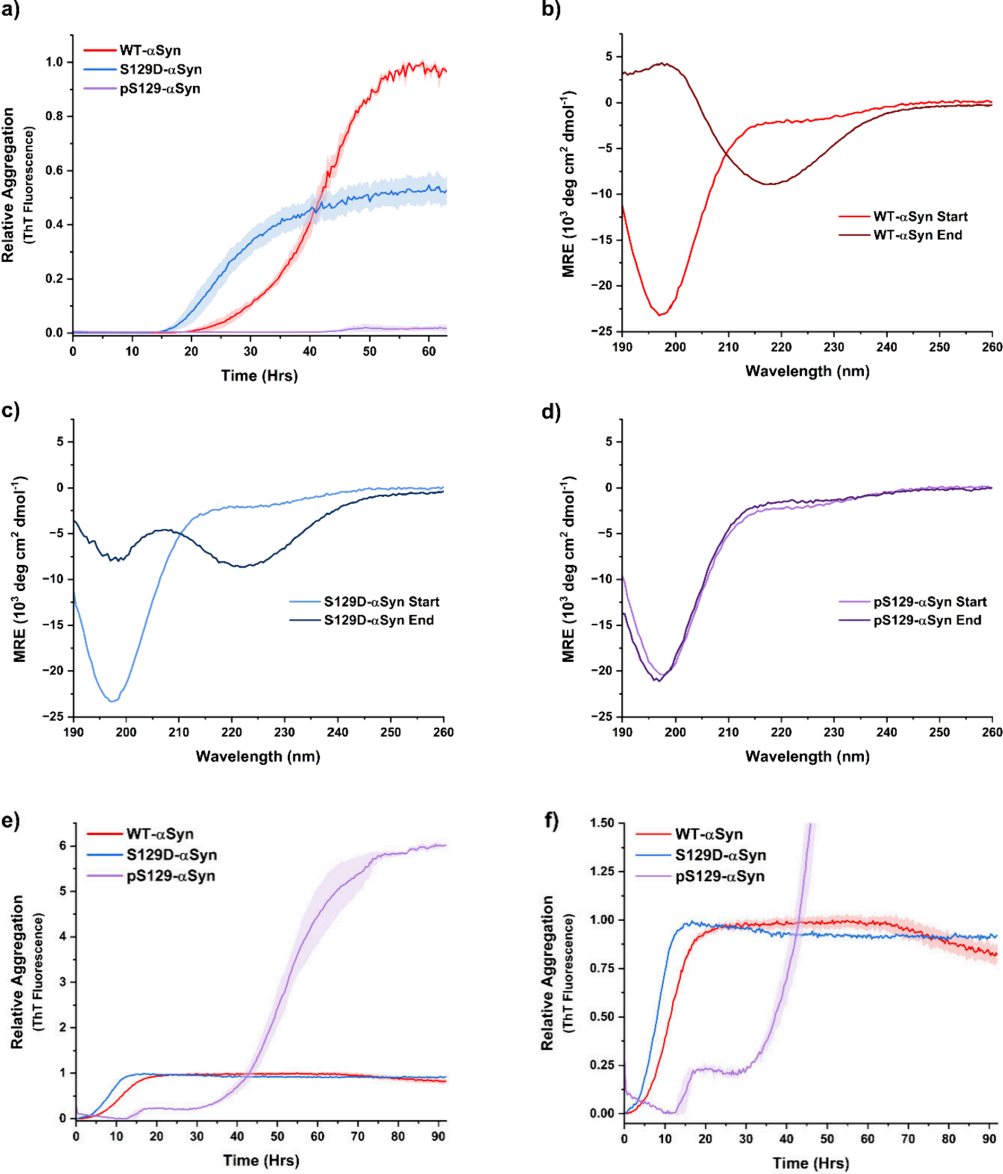
Aggregation kinetics and secondary structure of αSyn variants. (a) Agitation-induced aggregation of 100 μM wild-type (red), S129D (blue), and pS129-αSyn (purple) monitored by Thioflavin T (ThT) fluorescence. S129D aggregates most rapidly, wild-type reaches the highest plateau, and pS129-αSyn shows minimal aggregation under these conditions. (b–d) Circular dichroism (CD) spectra collected at 0 h (light traces) and 60 h (dark traces) for wild-type (b), S129D (c), and pS129-αSyn (d). Wild-type and S129D transition to β-sheet-rich spectra consistent with fibril formation, whereas pS129-αSyn retains a predominantly disordered profile. (e,f) Lipid-induced aggregation in the presence of 50 μM DMPS small unilamellar vesicles (SUVs). (e) ThT fluorescence traces show variant-specific aggregation kinetics, with pS129-αSyn displaying delayed onset relative to wild-type and S129D. (f) Zoomed in end point ThT intensities reveal that pS129-αSyn achieves a higher final fluorescence signal than both wild-type and S129D. Shaded regions represent mean ± s.e.m. from three independent experiments.

We subsequently assessed aggregation in a membrane-associated context using DMPS small unilamellar vesicles (SUVs), which mimic the anionic phospholipid environment encountered by αSyn within synaptic vesicles, where αSyn physiologically interacts and subsequently nucleates aggregation. In this setting, all αSyn variants formed aggregates, but with distinct kinetics and ThT end point intensities (Figure 3e,f). αSyn-S129D aggregated more rapidly than wild-type, whereas pS129-αSyn displayed delayed onset yet ultimately achieved the highest ThT intensity. These data indicate that authentic phosphorylation exerts context-dependent effects: it suppresses aggregation under agitation but enhances fibril formation in a lipid environment, suggesting that the functional role of pS129 depends on the molecular milieu. While ThT fluorescence can be influenced by fibril structure and aggregate quantity, the observed trends are supported by CD measurements, indicating consistent β-sheet structure formation across conditions. These observations are consistent with previous reports that S129 phosphorylation preferentially occurs in membrane-associated αSyn and that membrane engagement can modulate both phosphorylation dynamics and downstream aggregation behavior.^24^

To assess whether authentic phosphorylation at S129 produces functional consequences in cells, we first examined the effects of exogenous wild-type, S129D-αSyn, and pS129-αSyn on SH-SY5Y neuroblastoma cells stably expressing GFP-αSyn. Neither wild-type nor S129D-αSyn induced marked changes in cellular morphology or intracellular GFP-αSyn signal after 24 h (Figure 4a,b). In contrast, pS129-αSyn treatment resulted in a significant increase in intracellular GFP-αSyn fluorescence intensity in a subset of cells, quantified as elevated corrected total cell fluorescence (CTCF) relative to both wild-type and S129D variants (Figure 4c, Supplementary Figure 3). Because the distribution of single-cell intensities was non-normal (Shapiro–Wilk test), differences were evaluated using a Kruskal–Wallis test followed by Dunn’s post hoc correction, confirming a significant effect of pS129 treatment. We note that these experiments quantify cellular responses following exposure to extracellular αSyn species and do not directly assess intracellular uptake or persistence.

**Figure 4:**
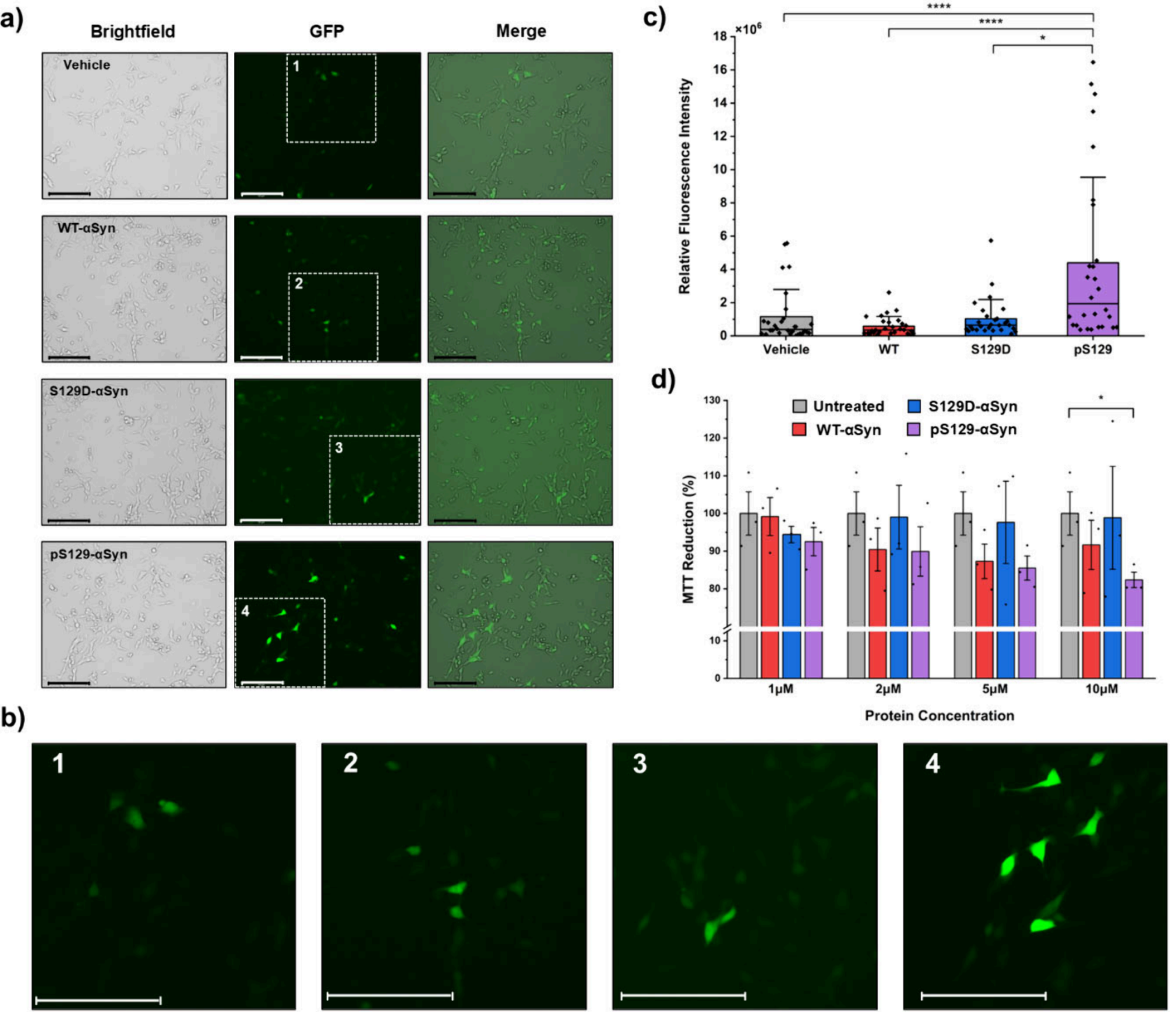
Cellular impact of wild-type, S129D and pS129 αSyn. (a) Representative images of SH-SY5Y neuroblastoma cells stably expressing GFP-αSyn following 24 h exposure to 10 μM exogenous wild-type, S129D-, or pS129-αSyn. pS129-αSyn induces a marked increase in intracellular GFP signal intensity in a subset of cells, consistent with altered αSyn processing or turnover. Scale bars = 125 μm. (b) Zoomed regions of GFP channel from (a). Scale bars = 125 μm. (c) Quantification of corrected total cell fluorescence (CTCF) from individual cells shown in (a). pS129-αSyn significantly increased intracellular GFP-αSyn signal relative to vehicle, wild-type, and S129D-αSyn. Because fluorescence values were non-normally distributed (Shapiro-Wilk test), differences were analyzed using a Kruskal–Wallis test (χ^2^(3) = 18.23, *P* = 3.9 × 10^–4^) with Dunn’s post hoc correction (*P < 0.05, **P < 0.01, ***P < 0.001, ****P < 0.0001). Bar charts represent mean + SD (*n* = 28 cells per condition, corresponding to the minimum number of analyzable cells across images, randomly selected from images with more than 28 cells) with the median indicated. (d) MTT viability assay in primary mouse cortical neurons treated with increasing concentrations (1–10 μM) of each αSyn variant for 96 h. pS129-αSyn produced the most pronounced reduction in metabolic activity, while wild-type αSyn showed more modest effects and S129D-αSyn exerted minimal effects across the same range. Each point represents an independent well using the same neuronal preparation, with mean ± s.e.m. shown.

To determine whether this altered intracellular behavior was associated with cytotoxicity, we next examined primary mouse cortical neurons exposed to each αSyn variant. As expected for monomeric αSyn preparations, overall toxicity by all variants was modest; however, both pS129-αSyn and wild-type αSyn produced a reproducible, dose-dependent reduction in metabolic activity, whereas S129D-αSyn elicited minimal effects across the same concentration range (Figure 4d). At 10 μM, pS129-αSyn induced a significant reduction in viability compared with both wild-type and S129D-αSyn (paired *t* tests across three independent preparations). Most notably, the phosphomimetic S129D consistently failed to reproduce the functional effects of authentic phosphorylation in either cellular model.

Together, these findings indicate that authentic Ser129 phosphorylation produces distinct cellular responses following exposure and neuronal toxicity profiles that are not captured by the S129D phosphomimetic, underscoring the importance of using chemically and structurally accurate post-translationally modified protein when examining αSyn function.

These observed cellular effects do not imply that S129 phosphorylation is intrinsically pathological. Rather, they demonstrate that authentic phosphorylation represents a functionally distinct biochemical state that cannot be inferred from phosphomimetic substitutions under identical conditions. Aggregate-mediated toxicity is well established but was not examined here, as this study focuses on chemically defined monomeric species.

We present a robust and scalable bacterial coexpression platform that enables authentic phosphorylation of αSyn at Ser129 by its native kinase, PLK2. This approach provides reproducible access to homogeneous, site-specifically modified protein without chemical ligation or semisynthetic steps, offering an efficient route to post-translationally modified intrinsically disordered proteins. The study examines chemically defined, monomeric αSyn variants and does not capture the full complexity of post-translational modification crosstalk, compartmentalization, or dynamic phosphatase activity present *in vivo*. In particular, S129 phosphorylation is reversible and regulated in neurons, and its functional consequences are likely to depend on cellular context, membrane engagement, and additional modifications.^16^

Using this system, we demonstrate that authentic phosphorylation exhibits biophysical and cellular outcomes fundamentally distinct from those of the commonly used S129D phosphomimetic. Our findings support the view that phosphorylation at S129 introduces a localized electrostatic change at the C-terminus that subtly modulates the conformational ensemble rather than inducing a discrete fold. Previous studies have similarly reported modest changes in local flexibility upon phosphorylation that are not faithfully reproduced by phosphomimetics.^18^ Because a phosphate is dianionic and possesses distinct geometry and hydrogen-bonding capacity relative to a carboxylate, even local differences are likely to influence how the C-terminal tail explores conformational space in ways that can either favor or oppose aggregation depending on context. These differences are summarized in Table 1.

**Table 1:**
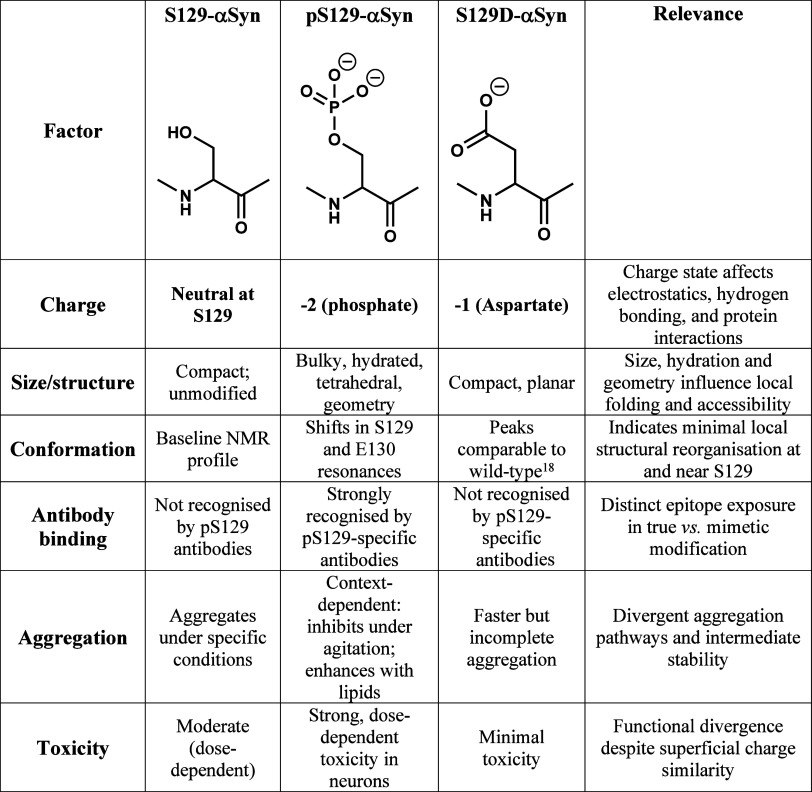
Comparison of Biochemical and Functional Properties of Wild-Type, Phosphorylated (pS129), and Phosphomimetic (S129D) αSyn: Summary of Charge, Structural, Biophysical, and Cellular Differences among αSyn Variants Modified or Substituted at Ser129^a^

a Parameters include phosphorylation state, NMR spectral features, antibody recognition, aggregation behavior, and cytotoxicity.

In line with this, multiple studies indicate that phosphorylation at S129 differentially modulates membrane association and can favor the emergence of structurally distinct fibril forms, altered seeding behavior or changes in toxicity profiles.^7,25^ The enhanced lipid-induced aggregation and increased neuronal toxicity we observe for pS129-αSyn are consistent with these reports and suggest that authentic phosphorylation biases αSyn toward bioactive assembly pathways that are not captured by phosphomimetic mutants. Together, these findings help reconcile long-standing inconsistencies in the literature and highlight pS129 as a chemically and functionally distinct αSyn state.^26,27^

## Materials and Methods

### pS129-αSyn Expression and Purification

Human wild-type-αSyn was subcloned into pACYDuet-1 and cotransformed into *E. coli* BL21 (DE3) with pET24a containing the human full length PLK2 gene (subcloned from the pDONR223-PLK2, which was a gift from William Hahn and David Root, Addgene plasmid #23691). Overnight starter cultures were grown in 2xYT with chloramphenicol (25 μg/mL) and kanamycin (50 μg/mL) at 37 °C, 200 rpm. For large scale expression, 2xYT flasks were inoculated to OD_600_ 0.1 and grown at 30 °C, 200 rpm until an OD_600_ 0.6–0.8. Expression was induced with 0.5 mM isopropyl β-d-1-thiogalactopyranoside (IPTG) and continued overnight at 16 °C, 200 rpm. Cells were harvested by centrifugation (JLA9.1000 rotor, 5000 rpm, 20 min, 4 °C) and resuspended in 20 mM Tris-HCl pH 8.0 with protease inhibitors (Abcam) before being lysed by five 30 s sonication cycles (Soniprep 150 plus, MSE) at maximum amplitude. Lysates were clarified by centrifugation (JA25.50 rotor at 18,000 rpm for 30 min at 4 °C). The soluble fraction was boiled for 15 min, recentrifuged (previous conditions) and subsequent supernatant then filtered (0.22 μm) before loading onto a 5 mL HiTrap Q HP column (Cytiva). The inclusion of anion exchange chromatography in the purification workflow is expected to reduce endotoxin contamination. The protein was then eluted on an ÄKTA Pure protein purification system (Cytiva) with a 0–60% gradient of 20 mM Tris-HCl pH 8.0 + 1 M NaCl over 13 column volumes. A final polishing and monomerization step was performed by SEC on a HiLoad 16/60 S75 SEC column preequilibrated with 20 mM sodium phosphate pH 6.5. Peak fractions were flash frozen and stored at −80 °C. All purification steps were analyzed by SDS-PAGE. Monomeric purity was verified by analytical SEC, with all αSyn variants eluting as single peaks, confirming the absence of oligomeric or aggregated species. The final protein sample was analyzed by ESI-QTOF LC/MS (Agilent). Results were analyzed by BioConfirm software (Agilent). All protein variants were expressed and purified in parallel using workflows to minimize variability between preparations, including potential endotoxin contamination.

### Wild-Type- and S129D-αSyn Expression

Initial starter cultures (2xYT) of *E. coli* BL21 (DE3) containing human wild-type- or S129D-αSyn constructs on pET21a were grown overnight in 2xYT with 100 μg/mL ampicillin (Amp) at 37 °C, 200 rpm. For large scale expression, 2xYT flasks were inoculated to OD_600_ 0.1 and grown at 37 °C, 200 rpm until an OD_600_ 0.6–0.8. Expression was induced with 0.5 mM IPTG and continued for 4 h at 37 °C, 200 rpm. Cells were harvested as before. Proteins were purified as previously described.^28^

### ^15^N-Labeled Wild-Type- and pS129-αSyn Expression

Overnight starter cultures (2xYT) were grown as above for *E. coli* BL21 (DE3) containing wild-type-αSyn on pET21a or wild-type-αSyn on pACYDuet-1 and PLK2 on pET24a. These were used to inoculate (OD_600_ 0.1) M9 starter cultures, prepared as described in Table S1. Following growth of 8 h at 37 °C, 200 rpm, a second M9 starter culture was then inoculated to OD_600_ 0.1 and incubated overnight at 37 °C, 200 rpm. M9 expression flasks (500 mL) were prepared (Supplementary Table 1) and inoculated to OD_600_ 0.1 and grown at 37 °C, 200 rpm to an OD_600_ 0.6–0.8. Protein expression was induced by 0.5 mM IPTG and continued overnight at 16 °C, 200 rpm. Cells were harvested as before. ^15^N labeled wild-type and pS129-αSyn were purified as previously described for unlabeled protein.^28,29^

### ^1^H-^15^N HSQC NMR

NMR experiments were performed on a Bruker Avance III HD 700 MHz spectrometer equipped with a 1.7 mm inverse triple resonance microcryocoil probe. ^1^H–^15^N TROSY spectra were carried out using the standard best TROSY pulse sequence from the Bruker library. NMR samples were dissolved in sodium phosphate (pH 6.5) at 100 μM and data collection was performed at 303 K with spectra referenced to H_2_O.

### Western Blot

3 mg of each protein was resolved by 4–20% Bis-Tris SDS-PAGE (mPAGE, Merck) and transferred to 0.45 μm Amersham Protran nitrocellulose membrane (Cytiva). Membrane was blocked by 5% (w/v) milk powder in TBS for 30 min at RT. Membranes were washed briefly with TBS-T before incubation with the primary antibodies (1:1000) in TBS-T + 1% milk overnight at 4 °C. Membranes were then washed with TBS-T and incubated with secondary antibody (1:2500) in TBST + 1% milk for 1 h at RT. Following washing with TBS-T and then TBS, bound antibodies were detected using Amersham ECL Western Blotting Detection Reagent (Cytiva). Western blots were imaged and quantified using the Fusion-SL Chemiluminescence System (Vilber Lourmat). Primary antibodies: Total αSyn antibody (CST #2628) or pS129-specific antibody (Abcam, EP1536Y). Secondary antibody: Goat anti-Rabbit IgG Peroxidase Conjugated (Merck, AP132P).

### Lipid Induced ThT Assay

1,2-Dimyristoyl-*sn*-glycero-3-phospho-l-serine (DMPS) (Avanti Research) was resuspended to 2 mM in 20 mM sodium phosphate buffer pH 6.5 by 3 h incubation at 45 °C, 1400 rpm. The solution was then freeze–thawed with dry ice five times, before being sonicated 5 × 30 s cycles with an amplitude of 10. A lipid-induced aggregation assay was then performed as previously described.^28^

### Agitation-Induced Aggregation Assay

Reactions (100 μL) containing 100 μM αSyn, 50 μM ThT, 0.05% sodium azide in 25 mM potassium phosphate pH 6.5, 100 mM potassium chloride, in 96-well half area polystyrene plates (Corning #3881) covered with sealing tape (Corning #6570) were collected in triplicate with a CLARIOstar microplate reader (BMG Labtech). A single glass bead of width 3 mm (VWR) was added to each well. The plate was shaken at 400 rpm for 30 s before each collection which occurred every 1200 s. CLARIOstar settings were: focal height set to 4.9 mm, gain set to 800, with an excitation filter of 440–15 nm and an emission filter of 480–15 nm, and a dichroic cutoff of 460 nm.

### Circular Dichroism

Start and end point samples were diluted to 10 μM in 20 mM sodium phosphate pH 6.5. CD spectra were collected using a Chirascan V100 (Applied Photophysics). Three scan sets were collected between 190 and 260 nm with a 0.5 nm bandwidth at 20 °C, in a 1 mm path length quartz cuvette. The scans were averaged and molar residue ellipticity (MRE) calculated.

### SH-SY5Y Cell Culture and Cell Imaging

The human neuroblastoma cell line SH-SY5Y overexpressing EGFP-labeled human SNCA (EGFP-αSyn-wild-type a gift from David Rubinsztein, Addgene plasmid #40822) was maintained in T75 flasks in complete media Dulbecco’s modified Eagle’s Medium/F-12 (ThermoFisher) supplemented with 10% fetal bovine serum, 100 units/mL penicillin-streptomycin (ThermoFisher), 2 mM l-glutamine (ThermoFisher) and 5% nonessential amino acids (ThermoFisher). Cells were maintained at 37 °C and 5% CO_2_ and passaged using 0.25% trypsin-EDTA (ThermoFisher) when they reached ∼90% confluency, to a maximum of 10 passages. αSyn variants diluted in PBS were added to final concentration of 10 μM (or PBS alone for vehicle control) and incubated for 24 hrs. Live imaging was performed using an EVOS M7000 imaging system. Corrected total cell fluorescence (CTCF) was calculated as previously described.^30^ The minimum number of fluorescently analyzable cells from within all fields of view (n = 28) was used for analysis. In fields containing more cells, 28 cells were randomly selected from the fluorescently visible population to ensure fair comparison. Single-cell GFP measurements were compared across conditions using a Kruskal–Wallis test with Dunn’s post hoc correction for multiple comparisons, as the data were not normally distributed (as determined by Shapiro-Wilk test).

### Primary Cortical Neurons

Neurons were cultured from CD1 mouse embryos at E15.5 as previously described^31,32^ and in accordance with UK Home Office Guidelines as stated in the Animals (Scientific Procedures) Act 1986, using procedures approved by the University of Bath Animal Welfare and Ethical Review Body. All tissue culture reagents were from Gibco unless stated otherwise.

Cortical tissue was collected by microdissection in PBS-Glucose (Dulbecco’s PBS, no calcium, no magnesium) supplemented with 6 mM d-(+)-glucose (Sigma-Aldrich) removing the striatum and the meninges and dissociated through trituration with a fire-polished pipet. Cells were resuspended in Neurobasal medium containing B27 supplement (ThermoFisher) 100 units/mL penicillin, 100 μg/mL streptomycin and 2 mM glutamine, seeded in Nunc treated multiwell plates precoated with 20 μg/mL poly-d-lysine hydrobromide (Sigma-Aldrich) and incubated at 37 °C, 5% CO_2_. Under these growth conditions at 6–8 days *in vitro* (DIV) cells had a well-developed neuritic network and were 98% β-tubulin III positive and <2% positive for the astrocytic marker GFAP.

### MTT Cell Viability Assay

A solution of 3-(4,5-dimethylthiazol-2-yl)-2,5-diphenyltetrazolium bromide (MTT) was prepared to 5 mg mL^–1^ in Neurobasal medium and added to each well containing primary cortical neurons to a final MTT concentration of 1 mg mL^–1^. The plate was incubated for 1 h at 37 °C, 5% CO_2_, and media removed. The formazan product was solubilized in 100% isopropanol and absorbance at 570 nm recorded using a CLARIOstar microplate reader (BMG Labtech). Three biological repeats were performed for each αSyn variant at each concentration. Because wild-type, S129D-αSyn, and pS129-αSyn were applied in parallel to the same preparation, paired *t* tests were used for comparisons between treatments.

## Supplementary Material



## Notes

Additional NMR data, uncropped SDS-PAGE and Western blot images, extended cellular imaging and quantification, and size-exclusion chromatography analysis of αSyn variants (PDF)
