# Supplementary material for: Authentic Phosphorylation of α‑Synuclein at Ser129 Reveals Functional Differences Not Captured by the S129D Phosphomimetic
Source: ACS Chem Biol. 2026 Apr 23;21(6):1251–9. doi: 10.1021/acschembio.6c00118 (PMC13288467; doi:10.1021/acschembio.6c00118)
Supplement: Supplementary file 1 [file cb6c00118_si_001.pdf]

Supplementary Information

Authentic Phosphorylation of Alpha-Synuclein at Ser129 Reveals Functional Differences Not Captured by the S129D Phosphomimetic

Scott G. Allen[1], Christopher Williams[2], Matthew P. Crump[2], Robert J. Williams[1] and Jody M. Mason[1]*

1. Department of Life Sciences, University of Bath, Bath, BA2 7AY, United Kingdom
2. School of Chemistry, University of Bristol, Bristol, BS8 1TS, United Kingdom

*To whom correspondence should be addressed: j.mason@bath.ac.uk
Keywords: Alpha-Synuclein, Parkinson's Disease, protein phosphorylation, Phosphomimetics, pS129, S129D

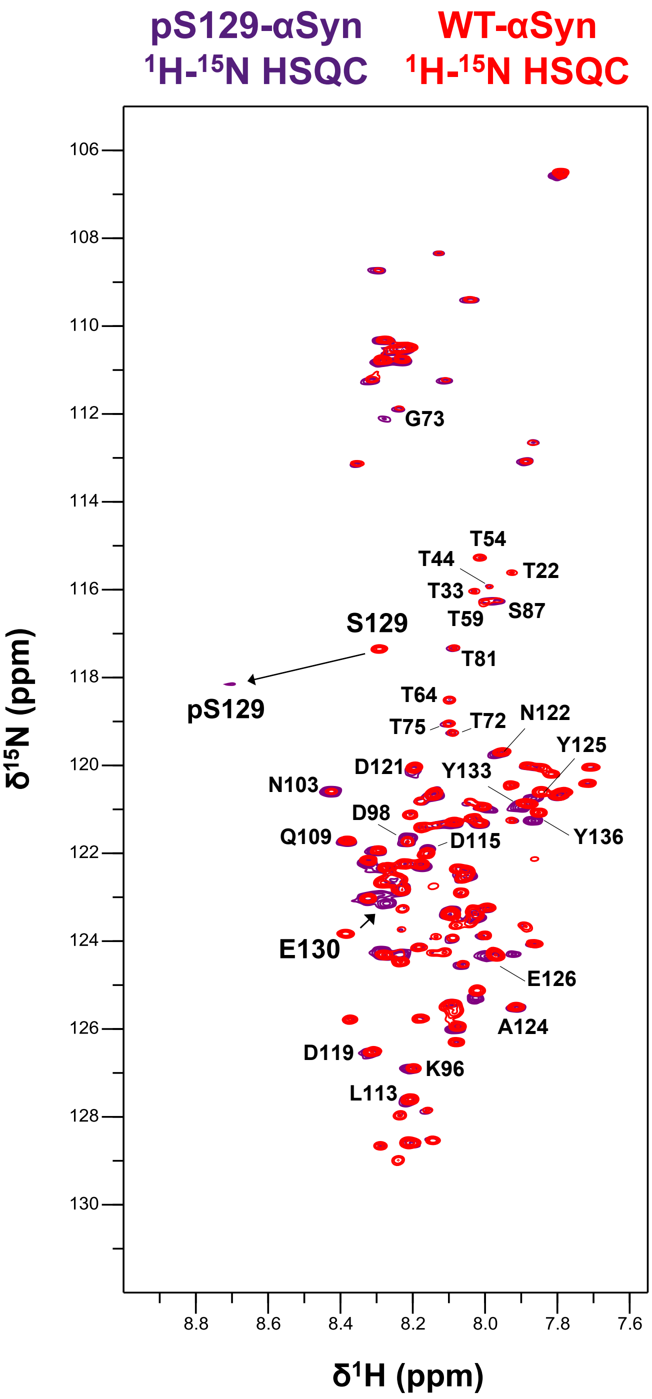

Fig S1. Overlaid ^{1}H–^{15}N HSQC spectra of ^{15}N-labelled wild-type αSyn and pS129-αSyn. A downfield shift of the S129 amide resonance (from ~8.3 ppm to ~8.8 ppm) and an upfield shift of E130 are consistent with site-specific phosphorylation at S129. Minor chemical shift perturbations are localised around the phosphorylation site (e.g. A124, E126, Y133). All other Thr and Ser residues (highlighted) show no detectable chemical shift changes, consistent with selective phosphorylation at S129.

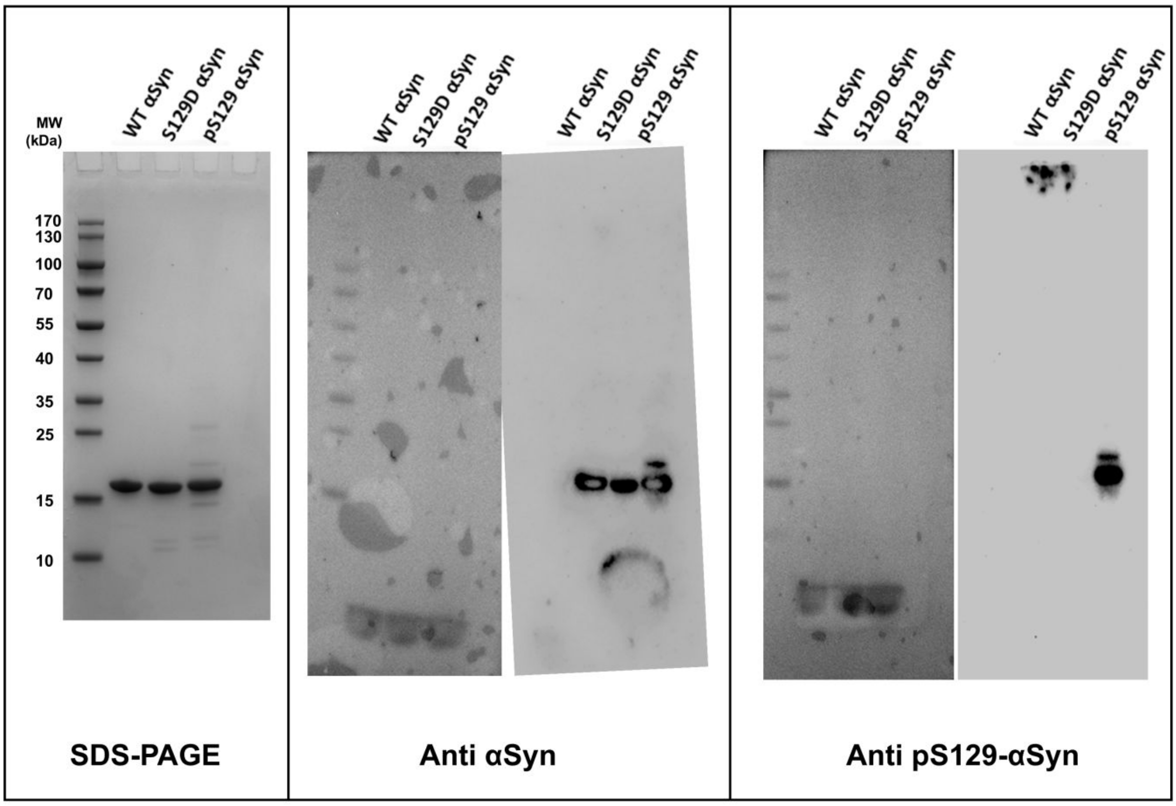

Fig S2: Full, uncropped SDS-PAGE and Western blot images for Figure 2c Uncropped originals corresponding to the gel/blot data in the main text. Left: SDS-PAGE showing total protein with molecular weight markers (kDa) indicated. Middle: Western blots probed with a pan-αSyn antibody (CST #2628). Right: Western blots probed with a pS129-specific antibody (Abcam EP1536Y). Lanes (left to right in each panel): WT αSyn, S129D αSyn, pS129 αSyn. An equal mass of each protein sample was loaded per lane, as per the Methods. Exposure differences reflect acquisition settings only; no lanes were removed or spliced. A minor higher-molecular-weight band is visible in the pS129-αSyn sample, which may reflect a low-abundance modified species or an electrophoretic artefact.

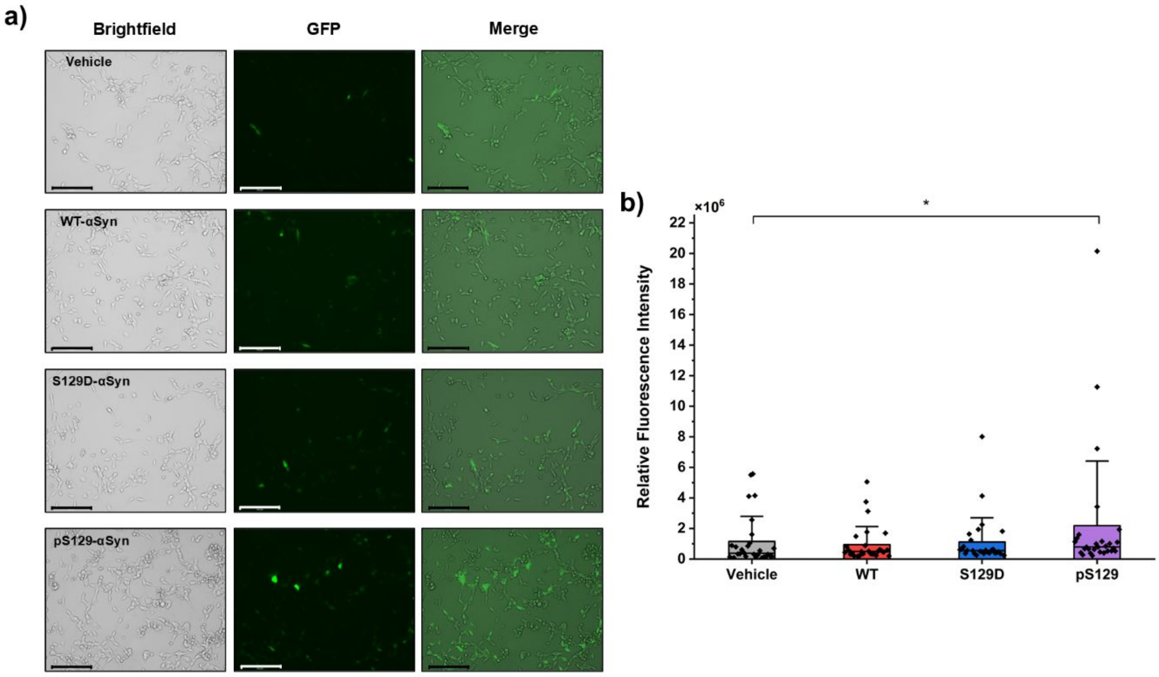

Fig S3: Cellular impact of exogenous αSyn variants. (a) Representative images of SH-SY5Y neuroblastoma cells stably expressing GFP-αSyn following 24 h exposure to 3.2 µM exogenous wild-type, S129D-, or pS129-αSyn. **(b)** Quantification of corrected total cell fluorescence (CTCF) from individual cells shown in (a). pS129-αSyn significantly increased intracellular GFP-αSyn signal relative to vehicle. Because fluorescence values were non-normally distributed (Shapiro–Wilk test), differences were analysed using a Kruskal–Wallis test with Dunn's post hoc correction. Bar charts represent mean + SD with median indicated from the minimum number of fluorescently active cells in all images (n = 28 cells per condition, corresponding to the minimum number of analysable cells across images, randomly selected).

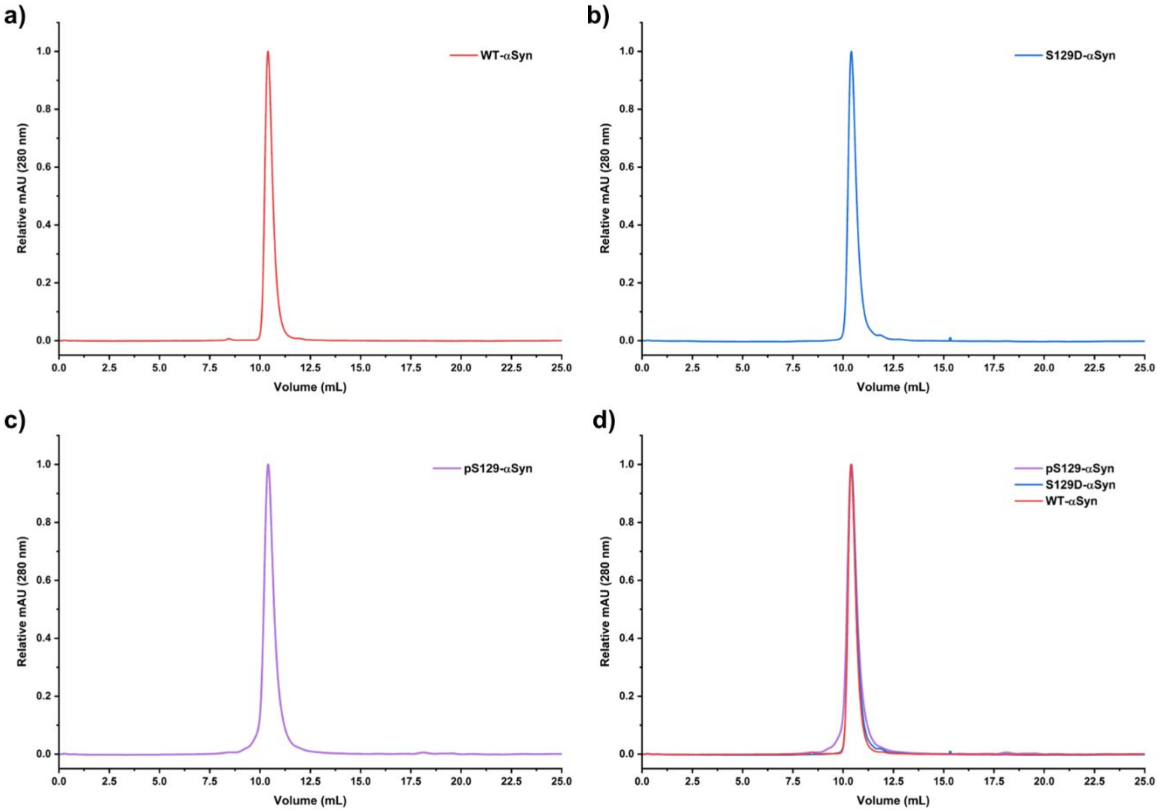

Fig S4: Size-exclusion chromatography confirms monomeric αSyn variants. **a-c)** SEC elution profiles of purified WT-αSyn, S129D-αSyn and pS129-αSyn in 20 mM sodium phosphate buffer, pH 6,5. All variants elute as single, symmetrical peaks corresponding to monomeric αSyn. **d)** Overlay of all three traces demonstrating identical monomer elution volumes and the absence of detectable higher-molecular weight species.

Table S1. Preparation of Media for ^{15}N Protein Labelling.

100x Trace Element Solution (per L)	5g EDTA, 0.83g $FeCl_3$, 84mg $ZnCl_2$, 13mg $CuCl_2$, 10mg $CoCl_2$, 10mg H_3BO_3, 1.6mg $MnCl_2$.
M9 Starter Culture Recipe (per 50mL)	0.56g 5x M9 Salts (Sigma Aldrich), 0.8mL 25% Glucose, 0.1mL 1M $MgSO_4$, 50µL 0.1mM $CaCl_2$, 50µL 100mg/mL Ampicillin.
M9 Expression Recipe (per 1L)	6g Na_2HPO_4, 3g KH_2PO_4, 0.5g NaCl, 0.5g ^{15}N NH_4Cl, 10mL 100x Trace Elements Solution, 20mL 25% Glucose, 2mL 1M $MgSO_4$, 1mL 0.1mM $CaCl_2$, 1mL 1mg/mL Biotin, 1mL 1mg/mL Thiamine, 1mL 100mg/mL Ampicillin.
